# The lipid-modulating effect of berberine in hyperlipidemic ApoE−/− mice

**DOI:** 10.3389/fphar.2026.1783475

**Published:** 2026-05-11

**Authors:** Naixin Shi, Xiaolei Zhou, Shengxian Wu, Yingying Zhang

**Affiliations:** 1 Beijing University of Chinese Medicine Dongzhimen Hospital, Beijing, China; 2 Zhengzhou Hospital of Traditional Chinese Medicine, Zhengzhou, Henan, China

**Keywords:** berberine, hyperlipidemia, lipid metabolism, sigma-1 probe technology, sigma-1 receptor

## Abstract

**Introduction:**

Berberine, a natural alkaloid from plants of the genus Coptis, shows significant lipid-lowering effects clinically. However, its exact mechanism in lipid metabolism is not fully understood. Sigma-1 receptor probes offer advantages such as strong *in vivo* applicability, non-invasiveness, high quantitative accuracy, and real-time visualization, which can enhance pharmacological research when combined with experimental validation.

**Objective:**

This study investigates the mechanism by which berberine improves lipid metabolism in ApoE−/− mice using Sigma-1 receptor probes.

**Methods:**

ApoE−/− hyperlipidemic mice were used as the model. Berberine treatment effects on lipid profiles were measured. Hepatic lipid metabolism was evaluated with Oil Red O staining. Sigma-1 receptor expression in mouse tissues was analyzed using [^18^F]FBFP probe, Western blot, and related technology.

**Results:**

After 6 weeks of treatment, berberine significantly reduced serum total cholesterol and low-density lipoprotein cholesterol levels, ameliorated hepatic lipid deposition, and upregulated the expression of hepatic Sigma-1 receptor in hyperlipidemic ApoE−/− mice.

**Conclusion:**

Berberine effectively improved dyslipidemia and hepatic lipid deposition in hyperlipidemic mice, which may be related to the modulation of hepatic Sigma-1 receptor expression, though the precise mechanism requires further validation.

## 1 Introduction

Hyperlipidemia, characterized by elevated levels of low-density lipoprotein cholesterol (LDL-C) and/or triglycerides (TG), is a lipid metabolism disorder and a significant risk factor for atherosclerosis and subsequent cardiovascular diseases (Abbasi et al., 2024; Pearson et al., 2021). Globally, about one-third of adults are affected by hyperlipidemia, contributing to a considerable proportion of coronary heart disease events and cerebrovascular accidents (NCD Risk Factor Collaboration NCD-RisC, 2020). This underscores the urgency of addressing this public health challenge. Effective control of hyperlipidemia is crucial for delaying the progression of cardiovascular and cerebrovascular diseases. A large-scale clinical study involving nearly 170,000 participants demonstrated that reducing LDL-C levels by 1.0 mmol/L can lower the annual incidence of major cardiovascular events by approximately 24% (Fulcher et al., 2015). However, existing lipid-lowering therapies are limited by adverse drug reactions and drug resistance, leading to suboptimal management of hyperlipidemia (Paraskevas et al., 2022).

Berberine (BBR), an isoquinoline alkaloid extracted from plants of the genus Coptis, has been widely confirmed in clinical studies to lower LDL-C, TG, total cholesterol (TC), and apolipoprotein B levels (Ju et al., 2018; Blais et al., 2023). Additionally, BBR exhibits unique effects in metabolic regulation, immune cell modulation, maintenance of cellular homeostasis, and gene expression, suggesting its potential as a safe and useful adjuvant therapy for diabetes, non-alcoholic liver disease, cancer, and cardiovascular diseases (Cheng et al., 2025; Asghari et al., 2025). Despite these documented therapeutic benefits, the molecular mechanisms through which BBR exerts its multi-target pharmacological effects remain incompletely understood. The Sigma-1 receptor (S1R), a chaperone protein located on the mitochondria-associated endoplasmic reticulum membrane, serves as a key regulatory node for cholesterol metabolism and protein secretion in the endoplasmic reticulum (Zhemkov et al., 2021a). Studies indicate that the antagonistic state of S1R can enhance the binding of proprotein convertase subtilisin/kexin type 9 (PCSK9) to S1R, potentially promoting the degradation of the low-density lipoprotein receptor (LDLR) and thus impairing lipid-lowering efficacy. Conversely, the agonistic state of S1R may enhance LDLR binding, stabilizing and protecting LDLR to favor lipid lowering (Zhao et al., 2023). Although direct evidence linking berberine to S1R activation is lacking, the overlapping downstream regulatory effects on pathways such as PCSK9 and LDLR suggest a mechanism warranting further exploration.

Leveraging the unique advantages of S1R probes, such as strong *in vivo* applicability, non-invasiveness, quantifiability, and real-time visualization, high-performance [^18^F]-labeled S1R molecular probes enable visual monitoring of quantitative changes in S1R expression during disease progression (Mastropasqua et al., 2025). Therefore, we employed [^18^F]FBFP and S1R probe technology to investigate the potential molecular mechanisms of berberine in treating hyperlipidemia, aiming to provide new experimental evidence and translational research directions for developing novel lipid-lowering drugs targeting the S1R.

## 2 Methods

### 2.1 Materials

#### 2.1.1 Experimental animals

For Experiment 1, 28 6-8-week-old male apolipoprotein E-deficient (ApoE−/−) mice served as the experimental model, with seven age-matched male C57BL/6J wild-type mice used as the control group. In Experiment 2, 45 ApoE −/− mice and 15 C57BL/6J wild-type mice of the same age and sex were employed. All animals were purchased from Beijing Huafukang Biotechnology Co., Ltd.

#### 2.1.2 Diet

The experimental diets were divided into two groups: a standard control diet and a high-fat diet. The high-fat diet (HFD) was formulated to contain 0.15% cholesterol, 21% fat, and 78.85% maintenance diet. Both diets were supplied by Beijing Huafukang Biotechnology Co., Ltd.

#### 2.1.3 Medications, reagents, and Instruments

Berberine tablets (0.1 g per tablet, 100 tablets per bottle) were purchased from Chengdu Jinhua Pharmaceutical Co., Ltd. Atorvastatin calcium tablets (Lipitor®, 20 mg per tablet, 7 tablets per box) were supplied by Pfizer Inc.

### 2.2 Methods

#### 2.2.1 Establishment of animal models, Grouping, and drug administration

This study was approved by the Ethics Committee for Animal Research of the First School of Clinical Medicine, Beijing University of Chinese Medicine (Approval No. 20–41).

The study consisted of two parts. In Experiment 1, after 1 week of acclimatization, the wild-type mice were fed a standard diet and served as the control group (Control, n = 7). ApoE−/− mice (n = 28) were fed a HFD and randomly divided into four groups (n = 7 per group). Treatments were administered once daily for the duration of the study as follows: (1) HFD group: received vehicle (water) via oral gavage; (2) HFD + BBR (p.o.) group: received berberine (150 mg/kg/day) via oral gavage; (3) HFD + BBR (i.p.) group: received berberine (5 mg/kg/day) via intraperitoneal injection; and (4) Atorvastatin (ATO) group: received atorvastatin (10 mg/kg/day) via oral gavage. Model induction and drug intervention were carried out simultaneously for 6 weeks. During this period, mouse body weight was recorded at the same fixed time point each week.

The doses of berberine for oral (p.o.) and intraperitoneal (i.p.) administration in this study were selected based on its pharmacokinetic properties. Previous studies have reported that the absolute oral bioavailability of berberine is extremely low in rats (0.36%), necessitating a relatively high oral dose (150 mg/kg) to achieve effective plasma levels (Liu et al., 2010). In contrast, intraperitoneal injection bypasses intestinal first-pass metabolism, resulting in significantly higher systemic exposure (Kheir et al., 2010). Therefore, a lower intraperitoneal dose (5 mg/kg) was sufficient to elicit a comparable pharmacodynamic effect. The reported equivalent dose ratio between oral and intraperitoneal administration ranges from 10- to 100-fold; the 30-fold difference used in the present study falls within this established range (Wang and Zidichouski, 2018; Gu et al., 2015).

In ApoE−/− model mice, the dosing range of atorvastatin is typically broad, with different doses applied for distinct experimental purposes. Lower doses (2.5–10 mg/kg/day) are commonly used to examine baseline lipid-lowering or anti-inflammatory effects. Based on previous studies (Gong et al., 2023; Liu et al., 2018; Hong et al., 2020), we selected a dose of 10 mg/kg/day of atorvastatin calcium as a robust positive control for evaluating the therapeutic efficacy of berberine. This dose is close to the clinically translatable range, effectively reducing serum lipid levels in ApoE−/− mice without inducing marked systemic toxicity.

Experiment 2, after a 1-week acclimatization period, C57BL/6J mice were fed a standard diet and used as normal controls. The 45 ApoE−/− mice were randomly divided into three groups: (1) ApoE−/−: normal diet + daily gavage of pure water; (2) ApoE−/− Model: HFD + daily gavage of pure water; (3) Treatment: HFD + daily gavage of BBR. The BBR dosage was calculated as described previously. Modeling and drug intervention were conducted simultaneously for 6 weeks. During this period, body weight was recorded weekly at a fixed time point.

#### 2.2.2 Measurement of serological parameters

At the end of the 6-week intervention, blood was collected from the internal carotid artery of each mouse (Experiment 1). After standing for 30 min at room temperature, the samples were centrifuged at 3000 rpm for 15 min at 4 °C. The resulting serum was aliquoted and stored at −80 °C until analysis. Lipid levels - including TC, TG, LDL-C, and very-low-density lipoprotein cholesterol (VLDL-C) - were measured using a Beckman Coulter AU-480 automatic biochemical analyzer.

#### 2.2.3 Preparation of liver frozen sections and oil red O staining

Liver tissues from mice in Experiment 2 were processed for Oil Red O staining. Following a 10-min fixation in 4% paraformaldehyde, the tissues were rinsed thoroughly with distilled water, then immersed in 60% isopropanol for 3 min. Staining was performed by incubating the tissues in a working solution prepared from 60 mL of Oil Red O stock solution and 40 mL distilled water for 30 min. After staining, the tissues were differentiated in 60% isopropanol and rinsed again with distilled water. Counterstaining was carried out with hematoxylin for 2 min, followed by a final distilled water rinse. The samples were then mounted with an aqueous mounting medium, air-dried in the dark at room temperature, and examined under a microscope.

#### 2.2.4 [^18^F]FBFP and Sigma-1 receptor probes

To assess S1R expression and distribution, organs and tissues from Experiment 2 were collected. The synthesis of [^18^F]FBFP was performed as follows: ^18^F-fluoride was produced via the ^18^O(p,n)^18^F nuclear reaction using a GE PETtrace cyclotron (16.5 MeV; GE Healthcare) and trapped on a PS-HCO_3_ anion-exchange cartridge. The trapped activity was eluted with a solution of TEAB (2 mg) in 1 mL of acetonitrile/water (70:30, v/v). The eluate was dried under argon at 110 °C, followed by co-evaporation with three 1.0 mL aliquots of anhydrous acetonitrile under argon at the same temperature.

The precursor (Compound 10, 1.5–2.5 mg) in 0.5 mL of DMF was then added, and the mixture was heated at 150 °C for 15 min. After cooling, 0.1 M ammonium formate containing 0.5% acetic acid (pH 4.2) was introduced. Purification was carried out by semi-preparative HPLC on a Phenomenex Luna C18(2) column (10 μm, 250 × 10 mm) with a mobile phase of 15% acetonitrile/85% 0.1 M ammonium formate +0.5% acetic acid (pH 4.2) at 5 mL/min. The product fraction (≈27 min) was collected, diluted with 10 mL of 4.2% NaHCO_3_ in 50 mL of deionized water, and loaded onto a Waters C18 Classic SepPak cartridge. The cartridge was washed with 10 mL of water, air-dried, and the product was eluted with 1 mL of anhydrous USP-grade ethanol. The eluate was mixed with 3 mL of USP-grade sterile saline in a 10 mL syringe, passed through a 0.22 μm sterile filter (MILLEX-GV), and dispensed into a degassed sterile vial containing 7 mL of sterile saline. Prior to intravenous injection, the formulated solution was analyzed by HPLC (Gemini NX column, 250 × 4.6 mm, 5 μm; mobile phase: 17% acetonitrile/83% 0.1 M ammonium formate +0.5% acetic acid, pH 4.2; flow rate 2 mL/min) to determine chemical purity, radiochemical purity, and specific activity. Identity of [^18^F]FBFP was confirmed by co-injection with an authentic FBFP reference standard.

#### 2.2.5 The expression levels of Sigma-1 receptor were analyzed by western blotting

Liver, scapular adipose tissue, peritoneal adipose tissue, and epididymal adipose tissue were harvested from mice in Experiment 2 for subsequent analyses. RIPA protein extraction buffer was prepared fresh and supplemented with protease inhibitors (and phosphatase inhibitors for phosphorylated protein analysis). Immediately before lysis, 0.1 M PMSF stock solution was added to a final concentration of 1 mM. Tissue samples were weighed, and lysis buffer was added at a 1:9 (w/v) ratio. Homogenization was performed on ice using a Fluka electric tissue homogenizer at 15,000 rpm, with three 10-s cycles interspersed with 10-s pauses. Following homogenization, samples were incubated on ice for 20 min and then centrifuged at 13,000 rpm for 20 min at 4 °C. The supernatant was carefully collected, aliquoted, and stored appropriately for downstream analysis.

The BCA working solution was prepared by mixing Solution A and Solution B at a 50:1 (v/v) ratio. Bovine serum albumin (BSA) standard solutions were serially diluted, and tissue protein samples were appropriately diluted with PBS. The protein samples were then combined with the BCA working solution at a 1:8 (v/v) ratio. After thorough mixing, the reaction was incubated at 37 °C for 30 min. Absorbance at 570 nm was measured using a microplate reader. Protein concentrations were adjusted with RIPA lysis buffer. After addition of 5× reducing sample buffer, the final protein concentrations were standardized to 4 mg/mL, 2 mg/mL, and 1 mg/mL, respectively. The samples were subsequently boiled for 5 min to achieve denaturation.

Western blotting was performed for target protein detection. A 12% resolving gel and a 5% stacking gel were prepared according to the molecular weight of the protein of interest. Protein samples (20 μg per well) were loaded and separated by electrophoresis under constant voltage: 90 V for ∼20 min (stacking) followed by 160 V (resolving), with the run terminated once the pre-stained protein marker had adequately migrated. After transfer, Ponceau S staining was used to assess transfer efficiency and confirm uniform protein distribution, and individual lanes were marked. The membrane was then completely submerged in 3% BSA-TBST and gently agitated at room temperature for 30 min.

The membrane was incubated with primary antibody diluted 1:1000 in 3% BSA-TBST for 10 min at room temperature, followed by overnight incubation at 4 °C. The following day, the membrane was equilibrated at room temperature for 30 min and washed five times with TBST (3 min per wash). It was then incubated with a horseradish peroxidase-conjugated goat anti-rabbit IgG (H + L) secondary antibody diluted 1:10,000 in 5% skim milk–TBST for 40 min at room temperature with gentle shaking. After six 3-min washes with TBST, the membrane was incubated with ECL reagent for 3–5 min and exposed to X-ray film for 10 s to 5 min. The film was subsequently developed for 2 min and fixed to finalize detection.

#### 2.2.6 Statistical analyses

Statistical analysis was performed using SPSS 23.0. Normally distributed data are expressed as mean ± standard deviation (mean ± SD). One-way Analysis of Variance (ANOVA) followed by Tukey’s *post hoc* test was used for inter-group comparisons when homogeneity of variance was satisfied. In cases of heteroscedasticity, Welch’s ANOVA with Dunnett’s T3 *post hoc* test was applied. Non-normally distributed data are presented as median and interquartile range [M (P25, P75)], and the Kruskal–Wallis test with Dunn’s *post hoc* multiple comparisons was employed. All tests were two-tailed, and a P-value < 0.05 was considered statistically significant.

## 3 Results

### 3.1 BBR ameliorates the levels of TC and LDL-C

Compared with the Control group, TC and LDL-C levels were significantly elevated in the HFD group (*P* < 0.0001), confirming the successful establishment of the hyperlipidemia model (Table 1). No other significant differences were observed in multiple comparisons among all groups; however, both the HFD + BBR and ATO groups showed a clear downward trend in TC and LDL-C. Thus, separate analyses were performed for the HFD, HFD + BBR, and ATO groups. Results demonstrated that compared with the HFD group, the HFD + BBR group exhibited significant reductions in TC (18.97%, *P* < 0.05) and LDL-C (22.12%, *P* < 0.05). Similarly, ATO administration led to significant decreases in TC (20.22%, *P* < 0.05) and LDL-C (20.81%, *P* < 0.05) compared with the HFD group (Table 2). Weekly monitoring showed no significant differences in body weight among the groups (Table 3).

**TABLE 1:** Alterations in blood lipid levels of mice across different groups ( $\overset{–}{x}$ ± SD).

Group	Control	HFD	^^^ ^a^ *P*	HFD + BBR	^^^ ^b^ *P*	HFD + BBR (i.p.)	^^^ ^c^ *P*	ATO	^^^ ^d^ *P*
n	6	6	-	5	-	5	-	5	-
TC (mmol/L)	2.59 ± 0.17	28.68 ± 2.96	<0.0001	23.24 ± 3.49	0.174	26.55 ± 3.68	0.949	22.88 ± 3.37	0.124
TG (mmol/L)	0.26 ± 0.12	0.60 ± 0.51	0.704	0.83 ± 0.43	0.989	0.90 ± 0.33	0.903	1.18 ± 0.74	0.761
LDL-C (mmol/L)	0.37 ± 0.05	9.36 ± 1.18	<0.0001	7.29 ± 1.07	0.1058	8.54 ± 1.62	0.970	7.35 ± 1.21	0.160
VLDL-C (ng/mL)	0.15 ± 0.03	0.12 ± 0.05	0.959	0.13 ± 0.05	>0.999	0.10 ± 0.07	0.970	0.10 ± 0.07	0.970

Statistical analysis confirmed that the data from all five groups were normally distributed. Descriptive statistics are presented as mean ± SD. Inter-group differences were analyzed using one-way ANOVA, with Tukey’s *post hoc* test when the assumption of homogeneity of variance was satisfied. In cases where homogeneity of variance was violated, Welch’s ANOVA, was applied, followed by Dunnett’s T3 test for *post hoc* multiple comparisons.

^a^ ^^^ *P*, HFD, vs. Control.

^b^ ^^^ *P,* HFD + BBR, vs. HFD.

^c^ ^^^ *P*, HFD + BBR (i.p.) vs. HFD.

^d^ ^^^ *P*, ATO, vs. HFD.

**TABLE 2:** Changes in TC and LDL-C levels in mice among the three groups ( $\overset{–}{x}$ ± SD).

Group	HFD	HFD + BBR	^^^ ^a^ *P*	ATO	^^^ ^b^ *P*
n	6	5	-	5	-
TC (mmol/L)	28.68 ± 2.96	23.24 ± 3.49	0.041	22.88 ± 3.37	0.029
LDL-C (mmol/L)	9.36 ± 1.18	7.29 ± 1.07	0.028	7.35 ± 1.21	0.033

Statistical analysis confirmed that all three datasets were normally distributed and met the assumption of homogeneity of variance. Between-group differences were assessed using one-way ANOVA, followed by Tukey’s test for post hoc multiple comparisons when statistical significance was observed.

^a^ ^^^ *P*, HFD + BBR, vs. HFD.

^b^ ^^^ *P,* ATO, vs. HFD.

**TABLE 3:** Changes in body weight of mice across groups ( $\overset{–}{x}$ ± SD).

Group	Week1	Week2	Week3	Week4	Week5	Week6	Week7
n	7	7	7	7	7	7	6
Control	23.66± 0.62	24.62± 0.25	25.04± 0.13	25.87± 0.12	26.30± 0.30	26.68± 0.17	27.22± 0.31
HFD	22.29± 0.66	23.47± 0.28	23.96± 0.27	24.85± 0.20	25.48± 0.21	24.06± 0.20	24.44± 0.24
HFD + BBR	22.50± 0.74	23.44± 0.20	24.00± 0.25	24.85± 0.21	25.47± 0.13	26.29± 0.31	27.21± 0.20
HFD + BBR (i.p.)	22.65± 0.80	23.57± 0.21	24.08± 0.31	24.94± 0.16	25.49± 0.13	25.99± 0.08	26.55± 0.29
ATO	22.58± 0.97	24.16± 0.34	24.66± 0.43	25.76± 0.22	26.52± 0.25	26.94± 0.24	27.85± 0.26

Statistical analysis indicated that the data from all five groups followed a normal distribution.

### 3.2 BBR attenuates hepatic lipid deposition in ApoE−/− model mice

**FIGURE 1:**
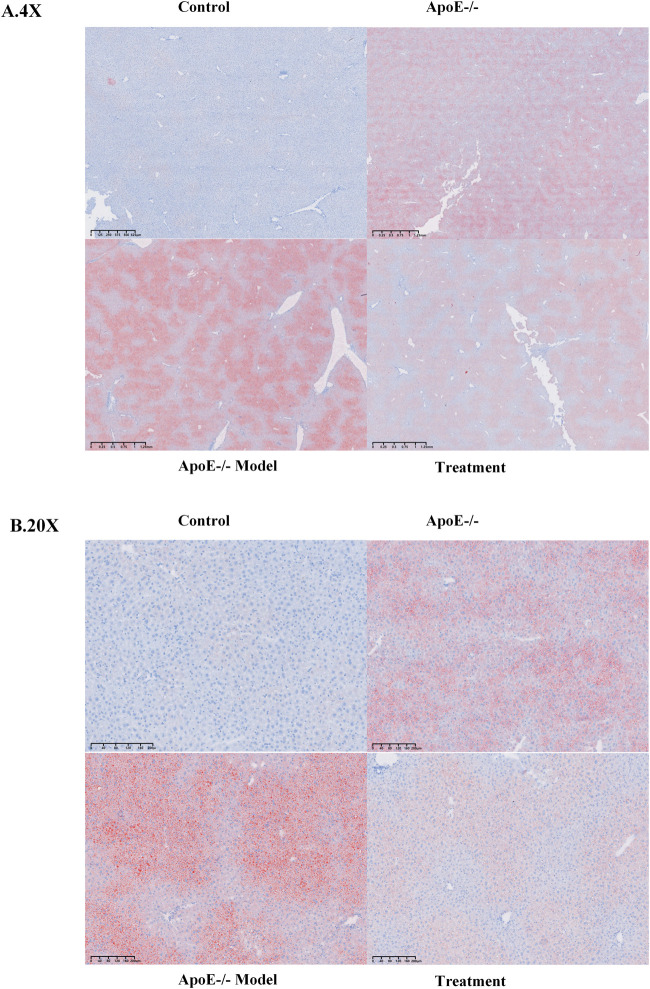
Oil Red O staining of mouse liver tissue. Note: **(A,B)** show representative micrographs of mouse liver sections stained with Oil Red O at magnifications of×4 and ×20, respectively.

Based on the Oil Red O staining analysis of liver tissues (Figure 1), hepatocytes in the C57BL/6J Control group exhibited minimal lipid droplet accumulation. In contrast, scattered focal orange-red lipid droplets were observed in the livers of ApoE−/− mice. Compared to the ApoE−/− group, the ApoE−/− Model group demonstrated severe, diffusely distributed lipid deposition accompanied by marked macrovesicular steatosis. Following BBR intervention, the accumulation of lipid droplets in hepatocytes of the treatment group was significantly reduced relative to the ApoE−/− Model group. These morphological findings suggest that BBR may help ameliorate high-fat-diet-induced hepatic lipid deposition in ApoE−/− mice.

### 3.3 Comparison of S1R expression among different groups of mice

**FIGURE 2:**
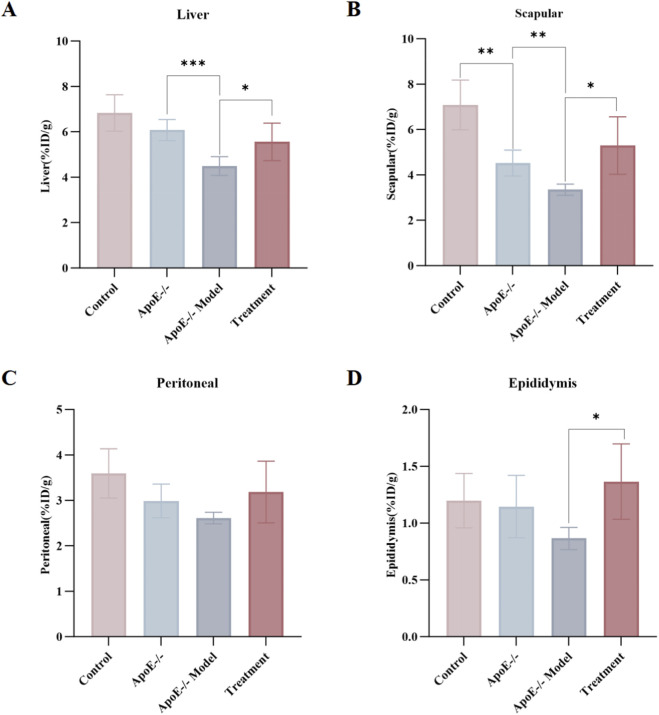
Expression of the S1R in Mice from Different Groups (Sigma-1 probe). Note: S1R expression was upregulated in the Treatment group. Notably, significant increases were observed in the liver **(A)**, scapular adipose tissue **(B)**, and inguinal adipose tissue **(D)** (*P* < 0.05). No significant elevation was detected in peritoneal adipose tissue **(C)** (*P* > 0.05).

To profile S1R expression across murine tissues, we employed the specific molecular probe [^18^F]FBFP (Table 4). Compared with ApoE−/− mice, ApoE−/− Model mice showed lower uptake of the S1R tracer (S)-[^18^F]FBFP in the liver (−26.05%), scapular adipose (−25.91%), peritoneal adipose (−12.67%), and inguinal adipose tissue (−24.51%). Following BBR treatment, tracer uptake increased markedly relative to the ApoE−/− Model group in the liver (23.73%), scapular adipose (57.87%), peritoneal adipose (21.94%), and inguinal adipose tissue (57.90%) (Figure 2). These results suggest that the therapeutic effect of BBR in this ApoE−/− hyperlipidemic model may be associated with the expression of S1R.

**TABLE 4:** Expression distribution of the S1R in different organs and tissues of mice ( $\overset{–}{x}$ ± SD).

Tissue	Control	ApoE−/−	^^a^P	ApoE−/− model	^^b^P	Treatment	^^c^P
n	8	7	-	8	-	8	-
Blood	0.29 ± 0.04	0.27 ± 0.02	0.569	0.29 ± 0.05	0.804	0.33 ± 0.18	0.967
Brain	7.87 ± 0.65	6.61 ± 0.47	0.003	7.31 ± 0.72	0.153	7.32 ± 0.59	>0.999
Heart	4.32 ± 0.38	3.14 ± 0.38	0.005	4.26 ± 0.79	0.007	3.97 ± 0.74	0.785
Liver	6.83 ± 0.81	6.08 ± 0.47	0.185	4.50 ± 0.42	0.0009	5.56 ± 0.83	0.018
Spleen	6.76 ± 0.66	6.47 ± 0.77	0.911	7.16 ± 0.91	0.425	7.82 ± 0.96	0.414
Lung	9.47 ± 1.90	9.08(6.53,11.69)	>0.999	10.30(7.76,10.75)	>0.999	9.27 ± 2.43	>0.999
Kidney	10.85 ± 0.81	8.89 ± 0.80	0.002	10.61 ± 0.94	0.006	10.66 ± 1.09	>0.999
Pancreas	21.18 ± 1.83	18.89 ± 2.51	0.336	17.41 ± 3.25	0.621	18.93 ± 2.10	0.617
Muscle	1.90 ± 0.15	1.44 ± 0.18	0.068	1.49 ± 0.23	0.994	1.79 ± 0.67	0.787
Bone	3.02 ± 0.85	2.04 ± 0.25	0.106	2.50 ± 0.30	0.033	2.49 ± 0.70	>0.999
Scapular	7.09 ± 1.10	4.53 ± 0.57	0.002	3.35 ± 0.24	0.006	5.30 ± 1.27	0.015
Peritoneal	3.60 ± 0.54	2.99 ± 0.37	0.130	2.61 ± 0.13	0.168	3.19 ± 0.68	0.225
Epididymis	1.20 ± 0.24	1.15 ± 0.27	0.999	0.87 ± 0.10	0.170	1.37 ± 0.33	0.018
Stomach	0.71 ± 0.22	0.59(0.54,0.74)	>0.999	0.65 ± 0.15	>0.999	0.75 ± 0.20	>0.999

Normally distributed data are expressed as mean ± SD. One-way ANOVA, followed by Tukey’s *post hoc* test was used for inter-group comparisons when homogeneity of variance was satisfied. In cases of heteroscedasticity, Welch’s ANOVA, with Dunnett’s T3 *post hoc* test was applied. Non-normally distributed data are presented as [M (P25, P75)], and the Kruskal–Wallis test with Dunn’s *post hoc* multiple comparisons was employed.

^a^ ^^^ *P*, ApoE−/− vs. Control.

^b^ ^^^ *P,* ApoE−/− Model vs. ApoE−/−.

^c^ ^^^ *P*, Treatment vs. ApoE−/− Model.

### 3.4 Effects of BBR on the expression of S1R in mouse liver and adipose tissues

**FIGURE 3:**
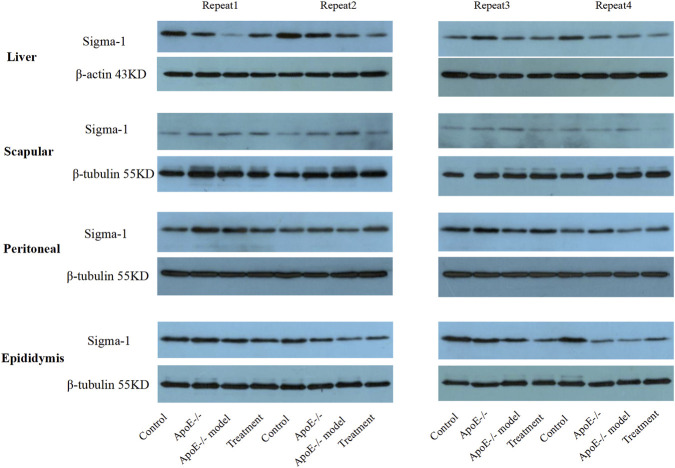
Expression levels of S1R in mice of each group (Western blot).

Based on the above studies, we examined S1R expression in various mouse tissues by Western blot. In liver tissue, S1R expression was downregulated in ApoE−/− Model group compared with Control group, and BBR treatment showed an upward trend relative to the ApoE−/− Model group (Figure 3). Comparison with the probe-based assay revealed a consistent trend only in the liver, whereas different expression patterns were observed in scapular, peritoneal, and epididymal adipose tissues. It should be noted, however, that the protein expression trend was only reproducible in the first experiment, and subsequent repeats did not yield consistent results. Thus, we tentatively hypothesize that BBR may exert its lipid-lowering effect by up-regulating S1R expression in the liver, though further evidence is required.

## 4 Discussion

Stroke affects approximately 600,000 individuals annually in China. Hyperlipidemia, a critical risk factor for stroke, severely compromises public health and quality of life. Currently, statins are the mainstay of clinical management for hyperlipidemia (Lewis and Segal, 2010). Although generally well-tolerated, statin use is associated with adverse effects such as elevated transaminase levels, gastrointestinal disturbances, myalgia, and even rhabdomyolysis, which remain significant clinical challenges (Karr, 2017). Therefore, there is an urgent need to explore safer and more effective therapeutic strategies to supplement or replace existing treatments. In this study, we observed that berberine significantly reduced serum TC and LDL-C levels, and attenuated hepatic lipid accumulation in hyperlipidemic mice. Given the hypothesis that the lipid-lowering mechanism of berberine may involve modulation of S1R expression, we employed S1R probe detection and Western blot analysis to test this hypothesis. While the evidence remains preliminary, these findings still provide a new perspective for investigating the lipid-lowering mechanism of berberine.

Previous studies have demonstrated that BBR can not only protect the gastrointestinal system (Liu et al., 2019) and inhibit cancer cell proliferation (Zheng et al., 2014; Li et al., 2017; Yang et al., 2013; Pan et al., 2017; Chen et al., 2015), but also modulate glucose and lipid metabolism (Sun et al., 2017; Liu et al., 2014), ameliorate non-alcoholic fatty liver disease (Guo et al., 2016), and exert significant lipid-lowering effects (Yang et al., 2022; Li et al., 2016; Sun et al., 2017). These mechanisms involve regulating key signaling molecules, such as AMP-activated protein kinase, sirtuin 1, PCSK9, LDLR, and nuclear factor kappa-B, as well as modulating the composition of gut microbiota (Feng et al., 2019). As the main carrier of cholesterol in human plasma, LDL is primarily metabolized via the LDLR in the liver. Thus, hepatic LDLR expression is a key determinant of circulating LDL-C levels (Cao et al., 2019). Research has shown that BBR enhances LDLR expression *in vivo* by upregulating LDLR mRNA and suppressing PCSK9 transcription (Kong et al., 2004; Li et al., 2009). However, the apparent contradiction between BBR’s low oral bioavailability, its low plasma concentration, and its pronounced *in vivo* pharmacological effects remains incompletely understood (Wang et al., 2017). Pharmacokinetic studies indicate that although BBR itself shows low bioavailability, its metabolites can reach substantial and sustained concentrations in the body (Maeng et al., 2002; Pan et al., 2002; Wang et al., 2005; Zuo et al., 2006). Some BBR metabolites have also been confirmed to upregulate LDLR and contribute to lipid-lowering (Zhou et al., 2014). These findings suggest that the lipid-lowering effect of BBR may not be mediated solely by the parent compound, but rather through the combined or even predominant action of its metabolites. Clarifying this mechanism is essential for understanding the true active components responsible for BBR’s efficacy.

In the present study, oral administration of BBR significantly reduced serum TC and LDL-C levels in hyperlipidemic mice, whereas intraperitoneal injection only showed a non-significant decreasing trend. Since intraperitoneal administration bypasses first-pass metabolism—increasing systemic exposure to the parent drug while reducing its hepatic metabolic conversion—these results indirectly support the hypothesis that BBR’s lipid-lowering effect may depend on hepatic first-pass metabolism and subsequent metabolite formation. To clearly distinguish the respective contributions of the parent BBR and its metabolites, future studies should measure concentrations of both the parent compound and its major metabolites in liver tissue and conduct dose-response and time-course analyses. Our results show that, in a hyperlipidemic mouse model, BBR at 150 mg/kg/day achieved comparable efficacy to atorvastatin at 10 mg/kg/day in lowering both TC and LDL-C. This provides strong direct evidence supporting the potential of BBR as an effective natural lipid-lowering agent. Given the distinct chemical, pharmacokinetic, and mechanistic profiles of the two compounds, future studies involving full dose-response experiments will help further evaluate the therapeutic and economic feasibility of BBR in clinical practice. Furthermore, extensive literature confirms the favorable safety profile of BBR. This highlights its potential for an improved balance between efficacy and safety. Future research could explore combination regimens of BBR with low-dose statins, aiming to achieve enhanced lipid-lowering effects while reducing statin dosage and associated side-effect risks. Such strategies may offer greater translational value for clinical management.

The S1R is widely expressed in both the central nervous system (e.g., hippocampus, hypothalamus) and peripheral organs (e.g., liver, kidneys) (Penke et al., 2018). Evidence suggests that cholesterol can directly induce S1R aggregation in the absence of other proteins, indicating a potential specific interaction between the two (Zhemkov et al., 2021b). Based on this, we hypothesized that the S1R may be involved in regulating cholesterol homeostasis, thereby mediating the lipid-lowering effects of BBR. To test this hypothesis, we performed *in vivo* imaging and quantitative analysis using an [^18^F]-labeled S1R molecular probe, combined with validation by Western blot. The results showed that hepatic S1R expression was significantly lower in high-fat diet-fed mice than in normal controls, while BBR treatment partially restored its expression. Consistent with this, Oil Red O staining demonstrated that BBR effectively reduced hepatic lipid accumulation. These findings suggest that hyperlipidemia is associated with downregulation of hepatic S1R expression, and that BBR intervention can partially reverse this change. Although the reproducibility of Western blot results requires further validation, this study preliminarily indicates a potential link between hepatic S1R expression and hyperlipidemia, and suggests that BBR may exert its lipid-lowering effects, at least in part, by upregulating the expression of this receptor. These results provide a new perspective for understanding the pharmacological mechanisms of BBR, implying that its action may partly depend on modulation of S1R-related signaling pathways.

This study has several limitations. First, the observed changes in probe signal reflect the “availability” of the S1R rather than its absolute expression level or functional status. This signal may be influenced by multiple factors, including competition with endogenous ligands, alterations in receptor localization, and nonspecific binding. Therefore, directly equating the signal with receptor expression or functional activity requires caution (Gromek et al., 2014). Second, although Western blot experiments were repeated four times, the reproducibility of the results remains suboptimal. Third, while this study reveals a correlation between S1R expression and the lipid-lowering effect of BBR, a causal mechanistic relationship has not been established. Future studies should employ receptor-specific antagonists or tissue-specific gene knockout approaches to further validate the necessity of the S1R in BBR-mediated lipid-lowering and to systematically elucidate its downstream signaling pathways. This will provide a more solid theoretical foundation for the precise clinical application of BBR.

## 5 Conclusion

Studies have shown that BBR not only significantly reduces TC and LDL-C levels, but also effectively alleviates hepatic lipid accumulation. Observations in mouse models suggest that the lipid-lowering and metabolic benefits of BBR may be associated with its influence on S1R expression. This study provides new experimental insights into the potential mechanism of BBR in lipid-lowering therapy and opens avenues for further investigation of its underlying actions. In addition to conventional *ex vivo* histological staining and Western blot analysis, this study also incorporated *in vivo* molecular probe imaging. These findings offer preliminary support for the future translational application of such probe technology in clinical populations.

## Data Availability

The original contributions presented in the study are included in the article/supplementary material, further inquiries can be directed to the corresponding authors.
